# Response strategies of five common warm temperate plant species to insect defoliation

**DOI:** 10.1186/s12862-024-02334-y

**Published:** 2024-12-03

**Authors:** Ning Wang, Qiang Li, Pan Wu, Shijie Yi, Hongliang Ji, Xiao Liu, Tongli He

**Affiliations:** 1 School of Advanced Agricultural Sciences, Weifang University, 5147 Dongfengdong Road, Weifang, 261061 China; 2 School of Geography and Tourism, Qilu Normal University, 2 Wenbo Road, Jinan, 250200 China; 3 Institute of Ecology and Biodiversity, School of Life Sciences, Shandong University, 72 Binhai Road, Qingdao, 266237 China; 4 School of Tropical Medicine, Hainan Medical University, Haikou, 571199 China; 5 Observation and Research Station of Bohai Eco-Corridor, First Institute of Oceanography Ministry of Natural Resources, Qingdao, 266061 China

**Keywords:** Leaf damage, Resistance, Tolerance, Warm temperate zone

## Abstract

Under the background of global climate change, climate warming has led to an increase in insect herbivory, which significantly affects the growth, survival, and regeneration of forest plants in the warm temperate zone of China. Plants can adopt defense responses to adapt to insect defoliation. Therefore, field experiments were conducted on five common warm temperate species, *Quercus acutissima*, *Quercus serrata*, *Quercus aliena*, *Quercus dentata*, and *Robinia pseudoacacia*. We measured the leaf traits of healthy trees and insect defoliated trees, to explore the response strategies of common species in warm temperate zones to insect defoliation. Our results showed that native species stored more carbon in extreme environments for survival rather than growth, while the alien species *R. pseudoacacia* tended to adopt active resource acquisition strategies and were more inclined towards growth. The content of tannins and flavonoids in the alien species *R. pseudoacacia* did not significantly increase after leaf damage, while the content of secondary metabolites such as tannins, flavonoids, and total phenols in the native species *Q. acutissima*, *Q. serrata*, *Q. aliena*, *Q. dentata* increased significantly after leaf damage. This indicated that compared to alien species, native species invested more resources in defense, which might reduce resource allocation for growth. Thus, the native *Quercus* species have stronger resistance than the alien species *R. pseudoacacia* after insect defoliation.

**Supplementary Information:**

The online version contains supplementary material available at 10.1186/s12862-024-02334-y.

## Introduction

Global warming may cause an increase in herbivory by insects, seriously affecting the structure and function of forest ecosystems [6, 23]. Defoliation by insects is a widespread forest disturbance driver, affecting global forest health and ecosystem dynamics [38]. In recent years, warm temperate forest ecosystems have generally shown phenomena related to insect interference, and have become one of the most vulnerable regions in global forest ecosystems [15].

In the co-evolution process of insects and plants, plants have evolved insect defoliation defense responses composed of many related traits, including chemical defenses [11, 12, 16, 32], plant structural traits [19], and compensatory mechanisms [43]. Among them, Secondary metabolites are one of the most direct connections between plants and insects, and the changes in secondary metabolites can be used as an evaluation of plant insect resistance (e.g., total phenols, tannins, and flavonoids). For example, a study measured the flavonoids concentration in the leaves of *Triadica sebifera* Small before and after being defoliated by insects, and found that plants defoliated by insects had higher flavonoids concentration than non-defoliated plants [41]. Flavonoids play multiple roles in plant protection, including screening of ultraviolet radiation and herbivore deterrence [1]. As tannins and total phenols also are important antiherbivore defenses that can interfere with herbivore digestion [3, 45].

Insect defoliation significantly reduces the photosynthetic leaf area, consequently, the production of non-structural carbohydrates (NSCs) is diminished [8, 43]. Trees depend on their stored NSCs, which include soluble sugars and starch, for growth and survival, particularly when photosynthesis is compromised [20, 26, 36]. When insect defoliation curtails carbon assimilation, trees must draw upon their NSC reserves to sustain metabolic functions and facilitate tissue repair. Nonetheless, many plants possess compensatory strategies to mitigate the impact of leaf loss [17]. For instance, Eyles et al. [14] observed that *Pinus radiata* can partially offset the loss of foliage by enhancing its photosynthetic capacity. Furthermore, previous research indicates that a plant’s nutritional status can influence the density of insect herbivores, with plants of higher nutritional quality being more prone to the detrimental effects of defoliation [31]. Nitrogen and phosphorus are vital nutrients for essential life processes and are key limiting factors in plant growth and development, playing a pivotal role in regulating various physiological functions within plants [9, 37]. Measuring lignin content after insect defoliation is crucial for understanding the plant’s defensive strategies and the impact of herbivory on plant cell wall dynamics.

Blossey and Nötzold have put forward the Hypothesis of Evolution of Increased Competitive Ability (EICA), which predicts that changes in selection due to herbivory selection may drive rapid evolution of non-native species through reallocation of resources from defense to growth and reproduction, thus become more competitive. For instance, Zheng et al. [46] observed that *Chromolaena odorata*, a non-native plant, rapidly shifts its resource allocation from defense to growth and reproduction once it has evaded its natural predators, leading to a demonstrated increase in its competitive capabilities. In the present study, we focused on five woody species—four native species, *Quercus acutissima* Carruth., *Quercus serrata* Thunb., *Quercus aliena* Blume., *Quercus dentat*a Thunb., and one non-native species, *Robinia pseudoacacia* L.—which are prevalent in the deciduous broad-leaved forests of North China [29, 30]. For instance, *Q. acutissima* and *Q. dentata* are known for their significant contributions to soil stabilization and carbon sequestration, respectively, while *Q. serrata* and *Q. aliena* provide essential habitat and food resources for a diverse array of wildlife. The non-native species, *R. pseudoacacia*, has acclimated significantly to the ecological landscape of North China, primarily due to its rapid growth and nitrogen-fixing capacity, which have notably enhanced soil fertility [28]. As a member of the Fabaceae family, *R. pseudoacacia* was introduced to China in the 1980s and has since been seamlessly incorporated into the native Fagaceae species, coexisting with *Q. acutissima*, *Q. serrata*, *Q. aliena*, and *Q. dentata* in a diverse range of forest compositions. Based on this, we hypothesized that: (i) following insect defoliation, different species would exhibit distinct carbon allocation strategies, and (ii) native *Quercus* species would allocate a greater proportion of their resources to defense compared to the non-native *R. pseudoacacia*. Understanding the carbon allocation and defense strategies of these species to insect herbivory yields insights into vegetation distribution dynamics and is crucial for the restoration of forests in northern China.

## Materials and methods

### Study site

**Fig. 1:**
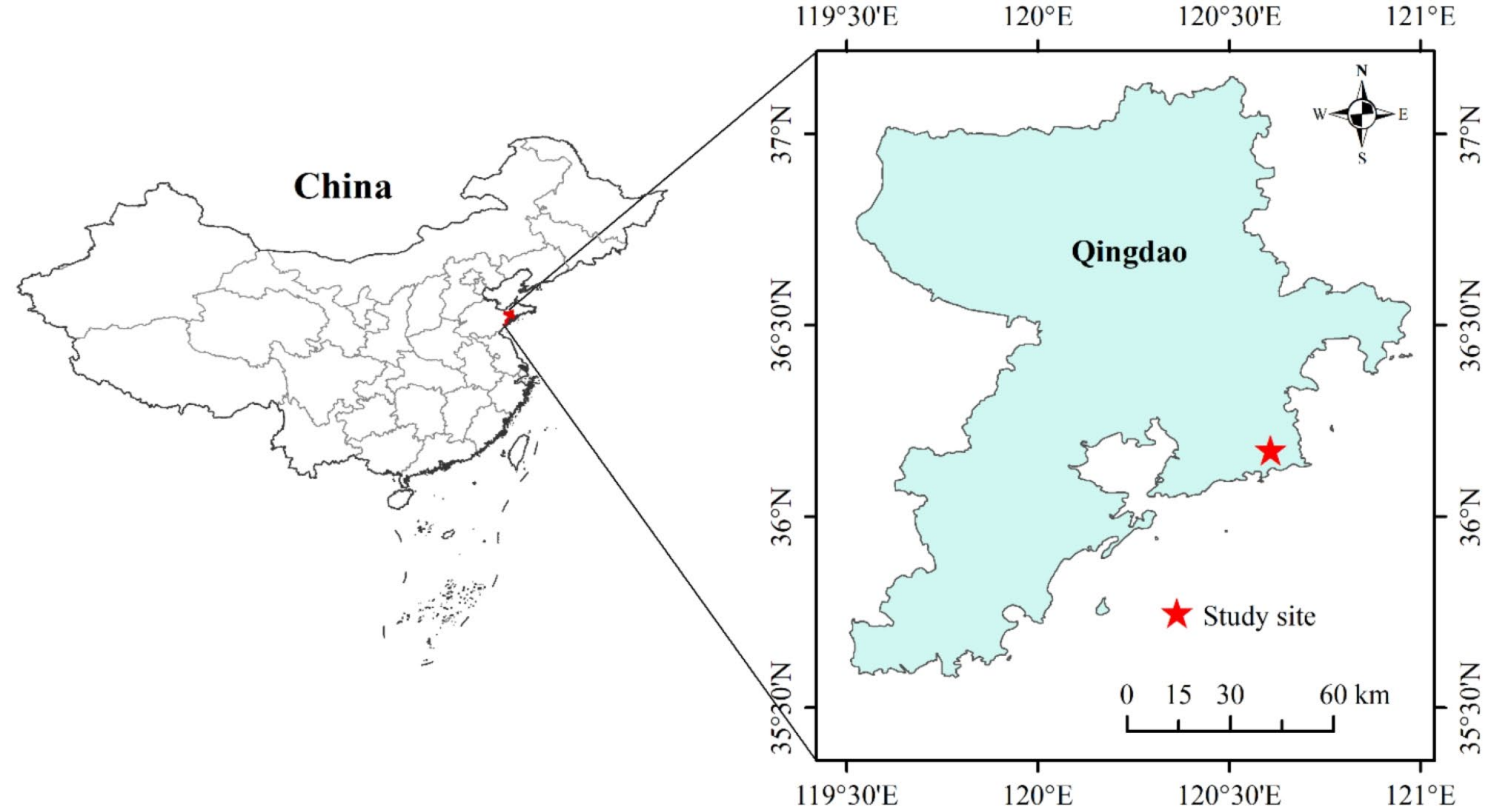
The map of study site

The study was performed at the Qingdao Forest Ecosystem National Positioning Observation and Research Station in Shandong province, China (Figs. 1 and 36°10’N, 120°37’E). This region is a typical representative area of warm temperate forests in China, with a climate type of warm temperate continental monsoon climate, an altitude of 389–700 m, an average annual precipitation of 900 mm, and an average annual temperature of 13℃. After conducting a preliminary vegetation survey in the station, it was found that plant species in the forest generally exhibit insect feeding, leading to a common phenomenon of leaf damage in the forest ecosystem (Fig. S1). Based on preliminary investigations, this study focuses on five common species in the sample plots of the research station: *Q. acutissima*, *Q. serrata*, *Q. aliena*, *Q. dentata*, and *R. pseudoacacia.* These species are the dominant species in the arbor layer across the warm temperate regions of Northern China [39]. In the study site, these species have been observed in sufficient numbers, where insect defoliation is a frequent occurrence.

### Experimental design

This study was conducted in September 2022 at the station. Based on a thorough investigation of the species composition, distribution, and insect defoliation in the wild, five representative and widely distributed common species in the warm temperate zone were selected. Each tree was randomly estimated the insect feeding area of 100 leaves. According to the percentage of the leaf area damaged per tree, the defoliation group (DE) had an insect feeding area greater than 50%, while the control group (CK) had an insect feeding area less than 5%. For each species, five individuals from DE group and five individuals from CK group were selected and labeled. After collection, we oven-dried leaves for 72 h at 75 °C, and stored the samples for subsequent quantification of non-structural carbohydrates content, lignin content, secondary metabolic content, nitrogen and phosphorus content. Leaves from each tree were pooled into a single sample for chemical analyses.

### Non-structural carbohydrates

Non-structural carbohydrates (NSCs), which primarily include soluble sugar (SS) and starch (ST), five replicates per treatment were analyzed using a standardized protocol. SS was extracted twice from 20 mg of dried tissue using 5 mL of 80% aqueous ethanol, with the extraction process conducted at a controlled temperature of 80 °C. The supernatants containing SS were then quantified by the anthrone-sulfuric acid method. Absorbance was measured at 620 nm using a spectrophotometer (UV-9000 S, Metash, Shanghai, China).

Following the SS extraction, the solid residue of each sample was extracted twice with HClO₄, maintaining a temperature of 60 °C during the process to ensure optimal extraction efficiency. After the extraction of ST, the absorbance at 620 nm was measured with the same spectrophotometer, again using the anthrone-sulfuric acid method. The contents of SS and ST, measured as glucose equivalents, were calculated for the dry mass (mg g^− 1^) of the tissue samples.

### Lignin

The determination of lignin content of five replicates per treatment was carried out using the acetyl bromide method. A 5 mg portion of dried plant sample was weighed for acetylation treatment. The acetylation process was conducted at a controlled temperature of 60 °C to ensure proper reaction efficiency. After the treatment, the UV absorption value of the acetylation solution was measured at 280 nm using a spectrophotometer. To ensure precise quantification, a standard curve method was employed. Based on the absorption value, the lignin content was calculated.

### Secondary metabolites

Secondary metabolites of five replicates per treatment were measured. Tannins exhibited pronounced UV absorption at 275 nm, and their quantification was achieved by leveraging the adsorptive properties of activated carbon, which selectively binds tannins. To quantify these compounds, standard curves were utilized. The sample was dried to a constant weight, pulverized, and sifted through a 40-mesh sieve. Approximately 50 mg of the powdered sample was then weighed and mixed with 1 mL of an extraction solution composed of 70% methanol and 30% water. This solution was sealed with a parafilm to prevent spillage and maintain the integrity of the extraction process. The mixture was subjected to extraction in a 70 °C water bath for 30 min, with intermittent shaking throughout the process to ensure thorough extraction. Following extraction, the sample was centrifuged at 12,000 rpm and 25 °C for 10 min. The supernatant was collected and diluted to a final volume of 1 mL with the extraction solution for subsequent analysis.

Under alkaline conditions, phenolic compounds reduced tungsten molybdate to form blue complexes with a characteristic absorption peak at 760 nm. The total phenolic content in the sample was assessed by quantifying the absorbance at 760 nm, employing the construction and application of standard curves to guide the analysis. The sample was dried to constant weight, crushed, and passed through a 40-mesh sieve. Approximately 100 mg of the sample was weighed and combined with 2.5 mL of a solvent mixture comprising 80% acetone and 20% water. This extraction solution was selected due to its ability to effectively extract tannins and other phenolic compounds from the plant material. The mixture was then subjected to ultrasonic extraction at 300 W power, with the sample being sonicated for 5 s with an 8-second interval between pulses, and the extraction was conducted at 60 °C for 30 min. After extraction, the mixture was centrifuged at 12,000 rpm and 25 °C for 10 min. The supernatant was then diluted to 2.5 mL with the extraction solution for measurement.

In an alkaline nitrite solution, flavonoids reacted with aluminum ions to produce a red complex with a characteristic absorption peak at 470 nm. For the quantification of flavonoids, standard curves were meticulously employed. The flavonoid content in the sample was quantified by measuring the absorbance of the extract at 470 nm. The sample was dried to constant weight, crushed, and sieved through a 40-mesh sieve. Approximately 0.1 g of the sample was weighed and mixed with 1 mL of an extraction solution composed of 70% methanol and 30% acetone. This solvent mixture was chosen for its ability to dissolve flavonoids and other water-soluble compounds efficiently. The mixture was then subjected to ultrasonic extraction at 300 W power, with a crushing duration of 5 s and an interval of 8 s, and the extraction was carried out at 60 °C for 30 min. After centrifugation at 12,000 rpm and 25 °C for 10 min, the supernatant was diluted to 1 mL with the extraction solution for analysis.

### Nitrogen and phosphorus

Nitrogen and phosphorus contents of five replicates per treatment were measured. Grind and sieve the harvested dry leaf samples, and determine the total nitrogen content (%) using the Kjeldahl method. Weigh about 0.2 g of the sample and place it in a digestion tube. Add 0.2 g of copper sulfate, 3 g of potassium sulfate, and 8 mL of concentrated sulfuric acid. Digest at 200 ℃ for 40 min, then heat up to 400 ℃ and digest for 40 min. Rinse the liquid in the digestion tube multiple times and pour it into a 50 mL volumetric flask to volume. Take 10 mL of the liquid into a clean digestion tube and place it in a K9860 nitrogen analyzer (Hanon, K9860, China) to measure the nitrogen content. Take another 5 mL of the test solution and transfer it into a 50 mL volumetric flask. Measure the total phosphorus content (%) using molybdenum antimony colorimetry and UV spectrophotometer (Shimadzu, UV-2550, Japan).

### Statistical methods

All variables were tested for normality (Shapiro-Wilk test) and homogeneity (Levene’s test) before analysis. Given the normal distribution and homogeneous variances of the data for both healthy and defoliated trees, an independent samples t-test was deemed suitable for examining the differences in leaf trait indicators between these two groups. Consequently, we employed this statistical method in SPSS 23.0 (IBM Corp., Armonk, NY, USA) to delve into the variations observed. Principal component analysis (PCA) was utilized to elucidate the interconnections among leaf traits. This analysis was executed within the R software environment (Oksanen et al. 2019). Before commencing the PCA, the data underwent meticulous preprocessing, which included centering the variables (subtracting the mean) and scaling them (dividing by the standard deviation) to ensure that each trait exerted a proportional influence on the subsequent analysis. Principal components were then selected based on their eigenvalues and the cumulative percentage of variance they explained, with the objective of extracting the most comprehensive information from the dataset. The results were ensured through the application of statistical tests that met the necessary assumptions and by the examination of data for outliers and influential observations.

## Results

### Treatment effects on carbon allocation

**Fig. 2:**
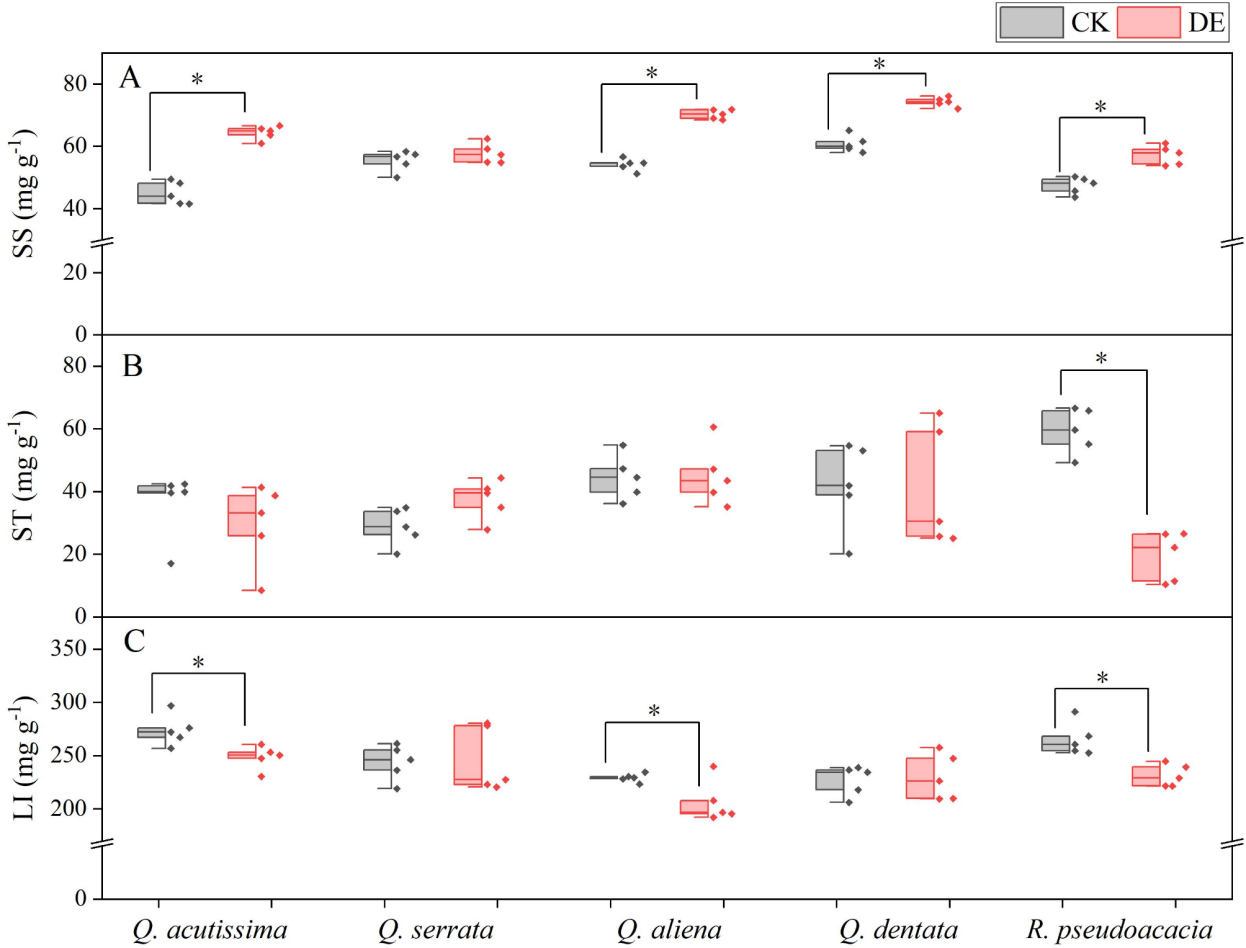
Soluble sugar content (**A**), starch content (**B**), and lignin content (**C**) of common species in warm temperate zones. * indicates significant difference between the control group and the defoliated group (*P* < 0.05). CK, control group; DE, the defoliated group. *n* = 5

After insect defoliation, the SS of *Q. dentata* was the highest among the five species, followed by *Q. aliena*, and there were differences in response among species (Table S2). Compared with the CK treatment, the DE treatment significantly increased the plants’ SS of *Q. acutissima* (CK: 45.02 ± 1.63, DE: 64.41 ± 0.98 mg g^− 1^), *Q. aliena* (CK: 54.18 ± 0.88, DE: 70.31 ± 0.68 mg g^− 1^), *Q. dentata* (CK: 60.85 ± 1.21, DE: 74.29 ± 0.67 mg g^− 1^), and *R. pseudoacacia* (CK: 47.47 ± 1.22, DE: 57.24 ± 1.39 mg g^− 1^) (Fig. 2A). However, of the five species examined, *R. pseudoacacia* exhibited the lowest ST (Table S2). Notably, only *R. pseudoacacia* showed a significant decrease in ST after insect defoliation (CK: 59.34 ± 3.28, DE: 19.45 ± 3.55 mg g^− 1^) (Fig. 2B). Following insect defoliation, the LI of *Q. aliena* was the lowest among the five species (Table S2). The DE treatment significantly decreased the plants’ LI of *Q. acutissima* (CK: 273.96 ± 6.59, DE: 248.57 ± 4.96 mg g^− 1^), *Q. aliena* (CK: 229.32 ± 1.82, DE: 206.58 ± 8.75 mg g^− 1^), and *R. pseudoacacia* (CK: 265.56 ± 6.97, DE: 231.38 ± 4.68 mg g^− 1^) compared with the CK treatment (Fig. 2C).

### Treatment effects on secondary metabolite

**Fig. 3:**
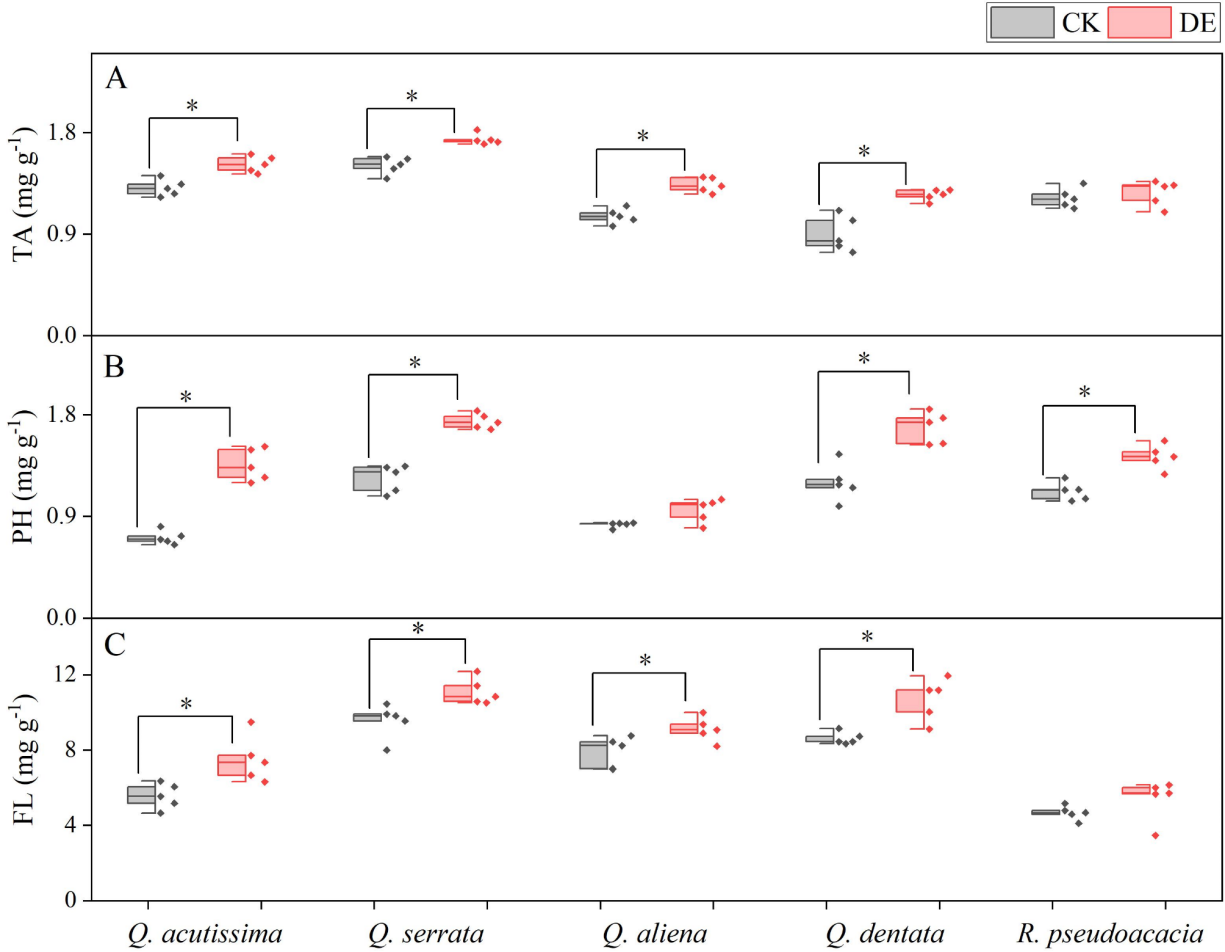
Tannin content (**A**), total phenolic content (**B**), and flavonoid content (**C**) of common species in warm temperate zones. * indicates significant difference between the control group and the defoliated group (*P* < 0.05). CK, control group; DE, the defoliated group. *n* = 5

Following insect defoliation, *Q. dentata* displayed the highest TA, PH, and FL among the five species (Table S2). The DE treatment significantly increased the seedlings’ TA and FL of four native *Quercus* species compared with the CK treatment (Fig. 3A, C). However, there was no significant difference in TA (CK: 1.22 ± 0.04, DE: 1.26 ± 0.05 mg g^− 1^) and FL (CK: 4.68 ± 0.17, DE: 5.41 ± 0.49 mg g^− 1^) of alien species *R. pseudoacacia* (Fig. 3A, C). As for plants’ PH, the DE treatment was significantly higher than the CK treatment of *Q. acutissima* (CK: 0.71 ± 0.03, DE: 1.36 ± 0.06 mg g^− 1^), *Q. aliena* (CK: 0.83 ± 0.01, DE: 0.95 ± 0.05 mg g^− 1^), *Q. dentata* (CK: 1.20 ± 0.07, DE: 1.69 ± 0.06 mg g^− 1^), and *R. pseudoacacia* (CK: 1.12 ± 0.04, DE: 1.43 ± 0.05 mg g^− 1^) (Fig. 3B).

### Treatment effects on nitrogen and phosphorus

**Fig. 4:**
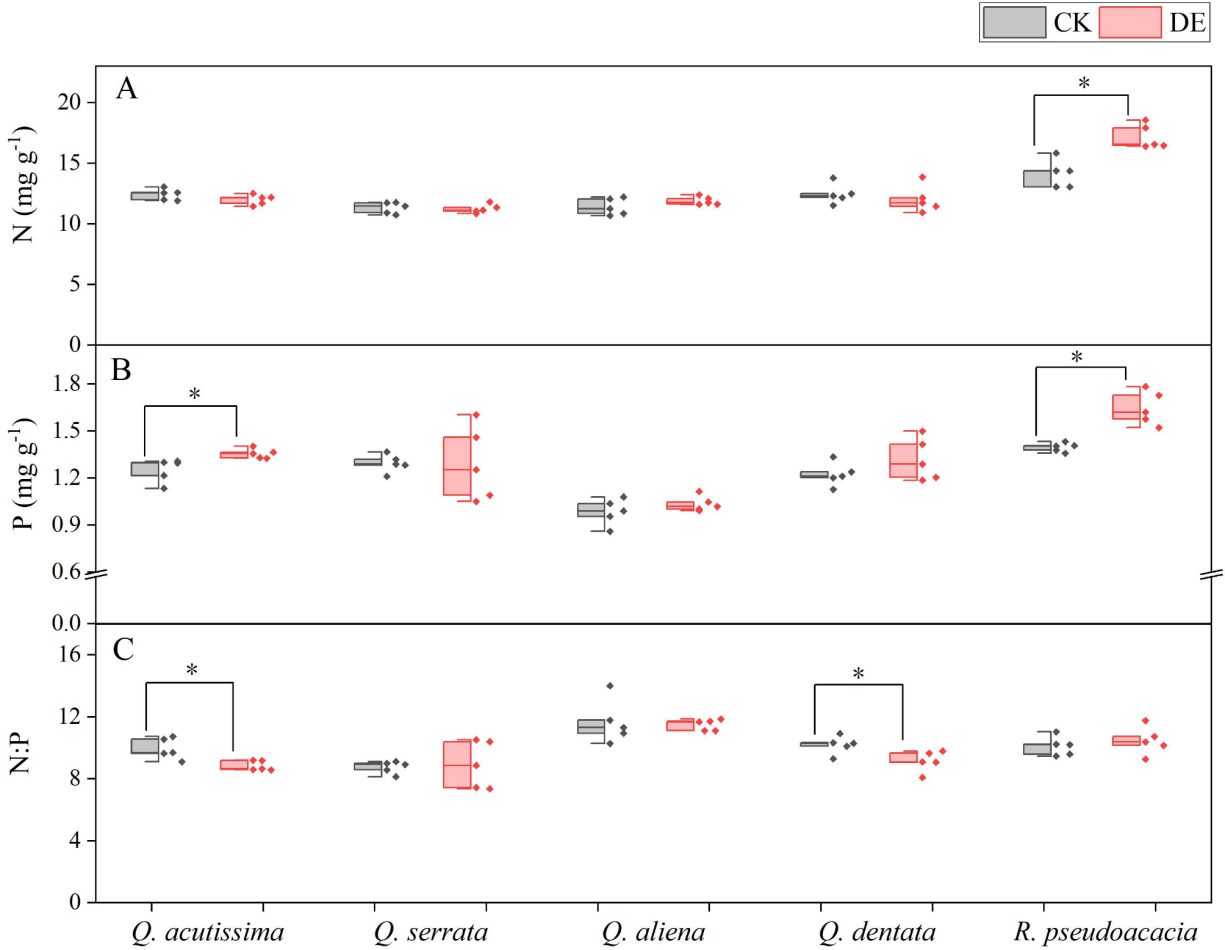
The nitrogen content (**A**), phosphorus content (**B**), and nitrogen-phosphorus ratio (**C**) of common species in warm temperate zones. * indicates significant difference between the control group and the defoliated group (*P* < 0.05). CK, control group; DE, the defoliated group. *n* = 5

Following insect defoliation, *R. pseudoacacia* exhibited the highest N and P among the five species assessed (Table S2). For the alien species *R. pseudoacacia*, we found that the N (CK: 14.13 ± 0.52, DE: 17.18 ± 0.45 mg g^− 1^) and P (CK: 1.40 ± 0.01, DE: 1.65 ± 0.05 mg g^− 1^) of the DE treatment were significantly higher than the CK treatment (Fig. 4A, B). After insect defoliation, the N: P of *Q. acutissima* (CK: 9.95 ± 0.30, DE: 8.85 ± 0.14 mg g^− 1^) and *Q. dentata* (CK: 10.19 ± 0.26, DE: 9.14 ± 0.30 mg g^− 1^) was significantly lower than the CK treatment (Fig. 4C).

### Principal component analysis

**Fig. 5:**
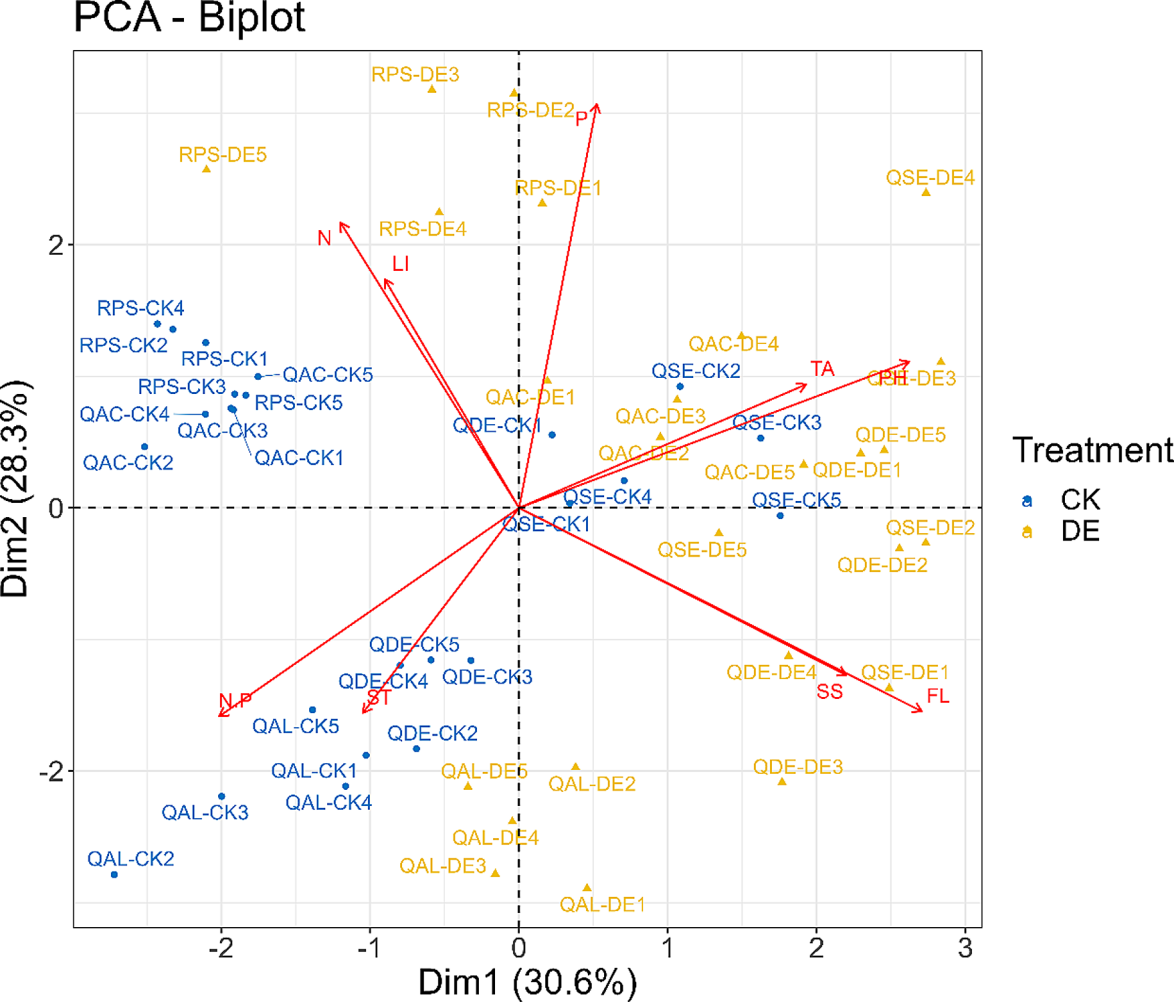
Principal component analysis (PCA) of traits of common species in warm temperate zones. CK, control group; DE, the defoliated group. *n* = 5

PCA based on leaf traits showed that the first two principal components could explain 58.9% of the total variance of leaf trait variation, with PC1 explaining 30.6% of the total variance and PC2 explaining 28.3% of the total variance (Fig. 5). The FL (0.814), PH (0.788), SS (0.661), and TA (0.580) have a significant contribution to the PC1 axis; P (0.923), N (0.652), LI (0.523), and N: P (-0.476) contribute significantly to the PC2 axis (Table S1). The CK treatment and the DE treatment of the alien species *R. pseudoacacia* showed significant differences in N and LI compared to other species. The DE treatment of four native *Quercus* species had significant differences in secondary metabolite compared with *R. pseudoacacia* (Fig. 5).

## Discussion

### Non-structural carbohydrate response to insect defoliation

Previous studies have reported that to overcome negative carbon balance, defoliation usually increases sugars allocation to the leaves [2, 13, 18]. Our results indicate that insect defoliation significantly increased leaf soluble sugars content of *Q. acutissima*, *Q. aliena*, *Q. dentata*, and *R. pseudoacacia* (Fig. 2A), which is consistent with previous reports. Moreover, in the present study, we observed that the starch content was reduced in the DE treatment of *R. pseudoacacia* (Fig. 2B), which indicated that starch was converted into soluble sugars to compensate for the lack of carbohydrates after insect defoliation [42]. *R. pseudoacacia* may possess a more efficient starch-to-sugar conversion pathway, which allows it to rapidly mobilize stored carbohydrates and maintain metabolic activity during times of stress. This could be due to the expression of specific enzymes or regulatory mechanisms that facilitate the breakdown of starch into soluble sugars. Meanwhile, we found that the lignin content of *Q. acutissima*, *Q. aliena*, and *R. pseudoacacia* were significantly lower than that of the control group after insect defoliation (Fig. 2C). The results suggested that plants reduced leaf structural investment by reducing leaf lignin content, thereby increasing soluble sugars content to achieve high carbon gain at a low leaf carbon cost. The PCA analysis also proved that there was a trade-off between leaf lignin content and soluble sugars content (Fig. 5). By reducing lignin content, plants may allocate more carbon towards NSC synthesis, which is crucial for maintaining metabolic processes, especially during periods of stress when carbon demands are high [44]. This strategic allocation of resources allows plants to achieve high carbon gain, balancing the need for structural integrity with the demand for rapid metabolic responses to stress.

### Differences in response strategies between native and non-native species after insect defoliation

The production and maintenance of plant defenses are energetically costly, and different species adopt different response strategies after insect defoliation [5]. Previous study showed alien plants under new selection pressures at the site of introduction will change their original growth defense strategies, shifting more resources from defense to growth and reproduction, thereby improving plant competitiveness [4]. In our study, insect defoliation significantly increased the seedlings’ tannins and flavonoids contents of four native *Quercus* species but did not show significant changes in the non-native species *R. pseudoacacia* (Fig. 3). Meanwhile, research has found that for different functional plant leaves, the nitrogen content in the leaves of fast-growing species is higher than that of relatively slower growing species. In this study, the nitrogen content of the alien species *R. pseudoacacia* was higher than that of the four native species of *Quercus* species. *R. pseudoacacia* belongs to fast-growing broad-leaved trees with a fast growth rate, while *Quercus* species mostly have a slow growth rate and a long growth cycle [33]. Similarly, in the insect feeding experiment of *Triadica sebifera*, it was found that its native population had higher resistance and lower tolerance, while the invasive population had lower resistance and higher tolerance, which was consistent with the results of this study [40]. In addition, principal component analysis of plant leaf traits also found that there were significant differences in tannin, flavonoid, and total phenolic content among the four native *Quercus* species after insect defoliation, while the alien species *R. pseudoacacia* showed no significant differences in tannin, flavonoid, and total phenolic content after leaf damage (Fig. 4). This indicated that compared to non-native species, native species would invest more resources in defense, which may reduce the allocation of resources for growth. The observed difference in *R. pseudoacacia* is a significant finding, but it is crucial to recognize that this conclusion is based on a single alien species. To strengthen the generalizability of these findings, future studies should compare *R. pseudoacacia* with a variety of native species and potentially include a broader range of alien species. This would provide a more comprehensive understanding of the resource allocation strategies employed by different species in response to environmental conditions.

Upon being subjected to insect defoliation, plants resort to utilizing carbohydrates to produce secondary metabolites, such as phenolic acids, which serve a defensive role, thereby improving their chances of survival [47]. The DE treatment notably augmented the tannins and flavonoids content in the seedlings of four indigenous *Quercus* species when compared to the CK treatment. However, this significant change was not observed in the non-native species *R. pseudoacacia* (Fig. 3A, C). Tannins, a type of phenolic compound with various resistive properties, can specifically bind to proteins [35]. A high concentration of tannins reduces the palatability of plants, imparting a bitter taste and deterring insect feeding, thus providing resistance against insect pests [10]. Flavonoids, on the other hand, exert a protective effect against insects by disrupting their normal metabolic processes [22]. The marked rise in secondary metabolite content in plants following insect defoliation suggests that native plants reallocate growth and defense resources post-insect feeding, with a greater emphasis on defense, thereby bolstering their defensive capabilities [25]. Moreover, this study revealed that the nitrogen and phosphorus content in *R. pseudoacacia* was significantly higher in damaged leaves compared to healthy trees (Fig. 3). Nitrogen and phosphorus are crucial elements influencing a plant’s photosynthetic capacity [24]. Nitrogen is vital for the synthesis of photosynthetic organs like chlorophyll [21], while phosphorus plays a pivotal role in plant metabolism, energy production, and protein synthesis [7, 27]. The enhanced nitrogen and phosphorus content in *R. pseudoacacia* leaves after insect defoliation can boost the plant’s photosynthetic capacity, allowing it to synthesize more carbohydrates, compensate for the loss of leaf area due to insect feeding, and maintain the stability of *R. pseudoacacia* [34]. Furthermore, the absence of direct photosynthesis measurements highlights a limitation in the study. To enhance the validity of this interpretation, future research should include direct measurements of photosynthetic rates. This would provide a more robust understanding of the impact of nutrient levels on plant photosynthetic potential and help to clarify the relationship between nutrient availability and photosynthetic capacity.

## Conclusions

In general, our study showed that insect defoliation leads to increased leaf soluble sugars content and reduced starch and lignin levels in both native and non-native species, indicating a shift towards carbohydrate conservation and enhanced photosynthetic capacity as a compensatory mechanism. Native species invest more in defense through increased secondary metabolites post-defoliation, while the non-native *R. pseudoacacia* prioritizes growth by maintaining higher nitrogen levels, reflecting distinct strategies for survival and recovery. Moving forward, it would be beneficial to integrate studies on both plant tolerance and resistance strategies in the aftermath of insect defoliation to gain a more thorough understanding of the diverse response strategies employed by plants to cope with insect herbivory.

## Electronic supplementary material

Below is the link to the electronic supplementary material.

Supplementary Material 1

## Data Availability

Data is provided within the supplementary information files.

## Abbreviations

CK: Control group
DE: Defoliated group
FL: Flavonoids
LI: Lignin
N: Nitrogen
NSC: Nonstructural carbohydrate
P: Phosphorus
PH: Total phenols
SS: Soluble sugar
ST: Starch
TA: Tannins
