# Supplementary material for: Response strategies of five common warm temperate plant species to insect defoliation
Source: BMC Ecol Evol. 2024 Dec 3;24:146. doi: 10.1186/s12862-024-02334-y (PMC11613790; doi:10.1186/s12862-024-02334-y)
Supplement: Supplementary file 1 — Supplementary Material 1 [file 12862_2024_2334_MOESM1_ESM.docx]

**
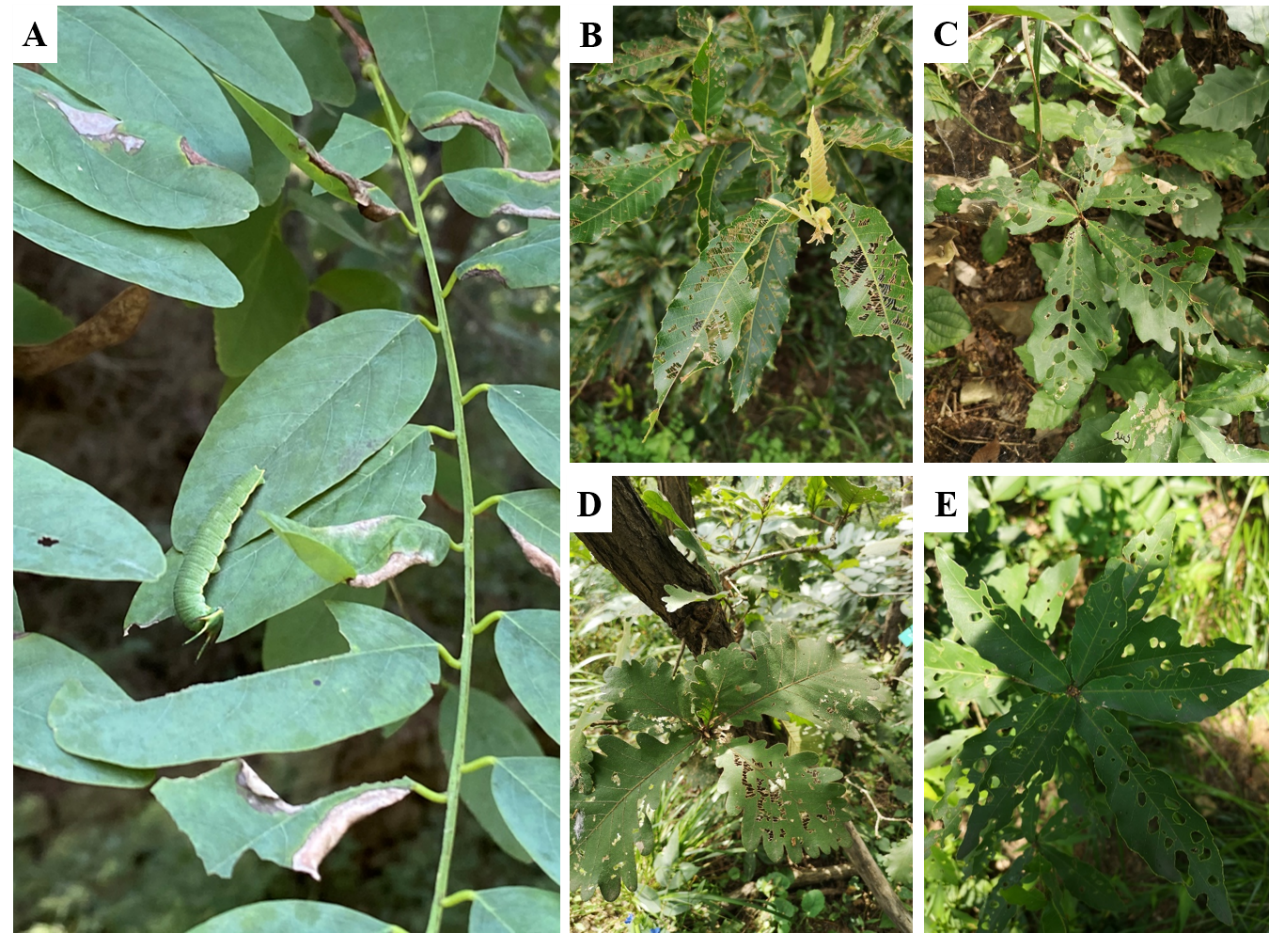
**

**Fig S1:** The phenomenon of leaf damage in warm temperate woody plants. A, *Robinia pseudoacacia*. B, *Quercus acutissima*. C, *Quercus aliena*. D, *Quercus dentata.* E, *Quercus serrata*.

**Table S1** Loading matrix of principal component analysis

| Parameters | PC1 | PC2 |
| --- | --- | --- |
| FL | 0.814 | –0.465 |
| PH | 0.788 | 0.334 |
| SS | 0.661 | –0.382 |
| N:P | –0.606 | –0.476 |
| TA | 0.580 | 0.282 |
| P | 0.157 | 0.923 |
| N | –0.361 | 0.652 |
| ST | –0.315 | –0.468 |
| LI | –0.271 | 0.523 |

FL, flavonoids; LI, lignin; N, nitrogen; P, phosphorus; PH, total phenols; SS, soluble sugar; ST, starch; TA, tannins.

**Table S2** Leaf traits (mean ± SE) of five plant species

| Species | Treatment | SS | ST | TA | PH | FL | LI | N | P | N:P |
| --- | --- | --- | --- | --- | --- | --- | --- | --- | --- | --- |
| *Q. acutissima* | CK | 45.02 ± 1.63 | 36.19 ± 4.80 | 1.31 ± 0.03 | 0.71 ± 0.03 | 5.57 ± 0.30 | 273.96 ± 6.59 | 12.42 ± 0.21 | 1.25 ± 0.03 | 9.95 ± 0.30 |
|  | DE | 64.41 ± 0.98 | 29.59 ± 5.88 | 1.52 ± 0.03 | 1.36 ± 0.06 | 7.52 ± 0.56 | 248.57 ± 4.96 | 11.99 ± 0.19 | 1.36 ± 0.01 | 8.85 ± 0.14 |
| *Q. serrata* | CK | 55.41 ± 1.49 | 27.15 ± 3.12 | 1.51 ± 0.03 | 1.24 ± 0.05 | 9.56 ± 0.41 | 243.81 ± 7.45 | 11.32 ± 0.21 | 1.29 ± 0.03 | 8.76 ± 0.18 |
|  | DE | 57.76 ± 1.43 | 37.55 ± 2.84 | 1.74 ± 0.02 | 1.74 ± 0.03 | 11.12 ± 0.31 | 246.06 ± 13.68 | 11.24 ± 0.16 | 1.29 ± 0.11 | 8.92 ± 0.69 |
| *Q. aliena* | CK | 54.18 ± 0.88 | 44.59 ± 3.21 | 1.06 ± 0.03 | 0.83 ± 0.01 | 7.90 ± 0.37 | 229.32 ± 1.82 | 11.41 ± 0.31 | 0.98 ± 0.04 | 11.67 ± 0.63 |
|  | DE | 70.31 ± 0.68 | 45.28 ± 4.44 | 1.34 ± 0.03 | 0.95 ± 0.05 | 9.12 ± 0.29 | 206.59 ± 8.75 | 11.89 ± 0.15 | 1.04 ± 0.02 | 11.50 ± 0.16 |
| *Q. dentata* | CK | 60.84 ± 1.21 | 39.80 ± 8.00 | 0.90 ± 0.07 | 1.20 ± 0.07 | 8.63 ± 0.15 | 226.87 ± 6.34 | 12.45 ± 0.37 | 1.22 ± 0.03 | 10.19 ± 0.26 |
|  | DE | 74.29 ± 0.67 | 41.14 ± 8.66 | 1.25 ± 0.02 | 1.69 ± 0.06 | 10.70 ± 0.50 | 230.29 ± 9.75 | 12.02 ± 0.50 | 1.32 ± 0.06 | 9.14 ± 0.30 |
| *R. pseudoacacia* | CK | 47.47 ± 1.22 | 59.34 ± 3.28 | 1.22 ± 0.04 | 1.12 ± 0.04 | 4.68 ± 0.17 | 265.56 ± 6.97 | 14.13 ± 0.52 | 1.40 ± 0.01 | 10.11 ± 0.28 |
|  | DE | 57.24 ± 1.39 | 19.45 ± 3.55 | 1.26 ± 0.05 | 1.43 ± 0.05 | 5.41 ± 0.49 | 231.39 ± 4.68 | 17.18 ± 0.45 | 1.65 ± 0.05 | 10.47 ± 0.41 |

CK, control group; DE, the defoliated group; FL, flavonoids; LI, lignin; N, nitrogen; P, phosphorus; PH, total phenols; SS, soluble sugar; ST, starch; TA, tannins.
